# Characteristics and Spatially Defined Immune (micro)landscapes of Early-stage PD-L1–positive Triple-negative Breast Cancer

**DOI:** 10.1158/1078-0432.CCR-21-0343

**Published:** 2021-06-09

**Authors:** Jodi M. Carter, Mei-Yin C. Polley, Roberto A. Leon-Ferre, Jason Sinnwell, Kevin J. Thompson, Xue Wang, Yaohua Ma, David Zahrieh, Jennifer M. Kachergus, Malvika Solanki, Judy C. Boughey, Minetta C. Liu, James N. Ingle, Krishna R. Kalari, Fergus J. Couch, E. Aubrey Thompson, Matthew P. Goetz

**Affiliations:** 1 Department of Laboratory Medicine and Pathology, Mayo Clinic, Rochester, Minnesota.; 2 Department of Public Health Sciences, The University of Chicago, Chicago, Illinois.; 3 Department of Oncology, Mayo Clinic, Rochester, Minnesota.; 4 Department of Health Sciences Research, Mayo Clinic, Rochester, Minnesota.; 5 Department of Health Sciences Research, Mayo Clinic, Jacksonville, Florida.; 6 Department of Cancer Biology, Mayo Clinic, Jacksonville, Florida.; 7 Department of Surgery, Mayo Clinic, Rochester, Minnesota.

## Abstract

**Purpose:**

Programmed death ligand 1 [PD-(L)1]-targeted therapies have shown modest survival benefit in triple-negative breast cancer (TNBC). PD-L1^+^ microenvironments in TNBC are not well characterized and may inform combinatorial immune therapies. Herein, we characterized clinicopathologic features, RNA-based immune signatures, and spatially defined protein-based tumor–immune microenvironments (TIME) in early-stage PD-L1^+^ and PD-L1^−^ TNBC.

**Experimental Design:**

From a large cohort of chemotherapy naïve TNBC, clinicopathologic features, deconvoluted RNA immune signatures, and intraepithelial and stromal TIME (Nanostring GeoMX) were identified in subsets of PD-L1^+^ and PD-L1^−^ TNBC, as defined by FDA-approved PD-L1 companion assays.

**Results:**

228 of 499 (46%) TNBC were PD-L1^+^ (SP142: ≥1% immune cells-positive). Using PD-L1 22C3, 46% had combined positive score (CPS) ≥ 1 and 16% had CPS ≥10. PD-L1^+^ TNBC were higher grade with higher tumor-infiltrating lymphocytes (TIL; *P* < 0.05). PD-L1 was not associated with improved survival following adjustment for TILs and other variables. RNA profiles of PD-L1^+^ TNBC had increased dendritic cell, macrophage, and T/B cell subset features; and decreased myeloid-derived suppressor cells. PD-L1+ stromal and intraepithelial TIMEs were highly enriched in IDO-1, HLA-DR, CD40, and CD163 compared with PD-L1-TIME, with spatially specific alterations in CTLA-4, Stimulator of Interferon Genes (STING), and fibronectin. Macrophage- and antigen presentation–related proteins correlated most strongly with PD-L1 protein.

**Conclusions:**

In this early-stage TNBC cohort, nearly 50% were PD-L1^+^ (SP142 companion assay) while 16% were PD-L1^+^ with the 22C3 companion assay. PD-L1^+^ TNBC had specific myeloid-derived and lymphoid features. Spatially defined PD-L1^+^ TIME were enriched in several clinically actionable immune proteins. These data may inform future studies on combinatorial immunotherapies for patients with PD-L1^+^ TNBC.

## Introduction

Triple-negative breast cancer (TNBC) is the most aggressive subtype of breast cancer and has limited targeted therapies. In the first-line metastatic setting, the addition of atezolizumab (anti–PD-L1 mAb) to nab-paclitaxel, improves progression-free survival (PFS) in patients with PD-L1^+^ TNBC (1), leading to its approval by the FDA in this setting. In the neoadjuvant setting, atezolizumab improves pathologic complete-response rates when added to chemotherapy (2). Similar results have been seen with the addition of pembrolizumab (anti–PD-1 mAb) to chemotherapy, which also improved PFS in the first-line metastatic PD-L1^+^ TNBC setting (for which it was recently granted FDA approval), and improved pathologic complete response rates in the neoadjuvant setting for early-stage TNBC (3–5). A tezolizumab and pembrolizumab were FDA-approved with nonequivalent PD-L1 companion assays (antibody clones and scoring systems; refs. 6, 7). The companion assay for atezolizumab uses the PD-L1 SP142 antibody clone and an assay cut-off point of ≥1% tumor-associated immune cells (IC), whereas pembrolizumab uses the PD-L1 22C3 pharmDx assay with PD-L1 positivity defined as a combined positive score (CPS) ≥10 (5). As most prior studies of PD-L1 expression in TNBC preceded the FDA approval of these companion assays—and used different antibody clones, assay cut-off points, or scoring systems—the reported frequency and features of PD-L1^+^ TNBC range considerably and have limited applicability for current PD-(L)1–targeted therapies in TNBC. Moreover, there is a paucity of data on the immune microenvironments, and potential actionable targets, in PD-L1^+^ TNBC as defined by these companion assays (8). In a well-characterized large cohort of patients with early-stage, chemotherapy-naïve TNBC (9), we evaluated the current FDA-approved PD-L1 SP142 and 22C3 assays to determine the frequency distribution and clinicopathologic features of PD-L1^+^ TNBC defined by these companion assays. As the survival benefits of immune checkpoint monotherapy have been modest in patients with advanced TNBC, there is growing interest in the use of novel combinatorial immunotherapies. We applied deconvolution methods for bulk RNA sequencing (RNA-seq) data to characterize the IC types and features of PD-L1^+^ (SP142) tumors. To complement this approach, we used high-plex digital spatial profiling (DSP, Nanostring GeoMX), a novel technology with a wide dynamic range, and micron-scale spatial resolution (10–13), to map and quantitate dozens of immune and other biomarker proteins in tumor immune microenvironments (TIME). Specifically, our goals were to (i) delineate spatially constrained PD-L1^+^ intraepithelial and stromal TIME and (ii) identify actionable targets to inform future studies of combinatorial immunotherapies for patients with PD-L1^+^ TNBC.

## Materials and Methods

This study was conducted in accordance with recognized ethical guidelines including the U.S. Common Rule and was performed after approval by Institutional Review Board (IRB). Written, informed consent was obtained from all subjects. A detailed clinicopathologic review of the Mayo TNBC cohort used in this study has been previously published (9). Briefly, the cohort is composed of 605 patients who underwent breast cancer surgery at Mayo Clinic, Rochester, Minnesota (1985–2012) with centrally-verified TNBC [defined as ER/PR <1% and HER2-negative per the 2013 American Society of Clinical Oncology (ASCO)/CAP guidelines], histologic subtyping and stromal TIL scoring (per recommendations of the International Immuno-Oncology Biomarker working group; ref. 14).

### PD-L1 scoring and companion assays

The PD-L1 SP142 assay [Ventana PD-L1 (SP142) Assay, Roche Diagnostics, performed in a CAP/Clinical Laboratory Improvement Amendments (CLIA)-certified clinical laboratory] was scored on 499 whole-sections of tumors from the TNBC cohort with available formalin-fixed paraffin-embedded (FFPE) material. Binary scoring (using the FDA-approved companion assay cut-point of PD-L1–stained tumor-infiltrating ICs, of any staining intensity, covering ≥1% of the tumor area; ref. 1) was performed by two board-certified anatomic pathologists with expertise in PD-L1 interpretation. For discordant cases, a final PD-L1 score was assigned by consensus following re-review. In addition, one reader performed exploratory binned scoring into categories of PD-L1^+^ IC and tumor cells. In addition, the PD-L1 SP142 and the PD-L1 22C3 assays were performed and scored into exploratory binned scores (one reader) on a previously constructed TNBC cohort-derived companion tissue microarray (TMA) composed of 1–2 × 1 mm tissue cores per FFPE tumor block (ref. 15; with *N* = 231 tumor cores scored with both assays). For the PD-L1 22C3 assay, the CPS method was used, defined as the number of PD-L1^+^ cells (ICs or tumor cells, with any staining intensity), divided by the total number of tumor cells, multiplied by 100 (3). For the TMA, tumors with > 1 scored tissue core with discordant scores were assigned the highest score.

### Statistical approach

Results from the Cox proportional hazards model are presented with HR and 95% confidence intervals (CI) in Table 2, including patients with known PD-L1 assay and adjuvant chemotherapy status (*N* = 404). The survival model includes established variables associated with recurrence-free and overall survival (OS) from Leon-Ferre and colleagues (9), age, plus those associated with PD-L1 (SP142) positivity (≥1% IC+) at *P* < 0.20. Correlation of PD-L1 and other pathologic characteristics are reported using Spearman correlation coefficient. Agreement between PD-L1 scores in TMA versus whole-slide was assessed by Cohen Kappa statistic for interrater agreement with 95% CIs (16).

### Bulk RNA-seq and cell-type quantification informatics

Bulk tumor RNA was extracted from 304 whole-section FFPE TNBC tumor specimens (HighPure RNA Extraction Kit) followed by TruSeq RNA Access library preparation and sequencing on a HiSeq2500. The RNA-sequencing data files were processed using the Mayo Clinic RNA-seq pipeline (MAP-RSeq; ref. 17), and then assessed utilizing the housekeeping genes (18), PPIA and SF3A1 for sample quality, and dfArray (19) for outlier identification. Conditional quantile normalization was applied to the gene expression data, and batch corrections were applied using the SVA combat algorithm (Supplementary Data S1; refs. 20, 21). A subset of 234 samples had both bulk tissue RNA-seq data and the whole-slide PD-L1 SP142 assay data (Supplementary Fig. S1). The normalized and scaled RNA-seq data for these 234 samples were submitted to the xCell web tool, a computational deconvolution method to assess the microenvironment contributions from transcriptome data (22). The xCell tool provided enrichment scores for 64 cell types, predominantly representing four specific immune-based or other lineages, as well as three enrichment scores. Tests for associations between PD-L1 and xCell features were calculated using the Kruskal–Wallis rank-based test for two groups (23), adjusted for multiple testing by FDR (24).

### High-plex digital spatial profiling

With the Nanostring GeoMX platform (Nanostring Technologies), the previously constructed companion TMA made from the TNBC cohort was evaluated using reagent panels composed of 58 oligonucleotide-tagged antibodies generated against immune or other biomarkers of interest, housekeeping proteins, and negative controls (Supplementary Data S2). For digital profiling, briefly, a 600-μm diameter circular region-of-interest per 1-mm tissue core was divided into a cytokeratin (CK)^+^ intraepithelial tumor segment (labeled with anti–pan-CK tagged with Alexa Fluor 532, NanoString) and adjacent CK^−^/SYTO13^+^ stroma segment. The ROI selection was blinded to the PD-L1 status of the tissue cores. Afterward, the intraepithelial and stromal segments were defined as PD-L1^+^ only if both the SP142 score for the core was ≥1% IC+ (with visual confirmation), and PD-L1 protein levels by DSP were greater than 2 fold background levels. A detailed description of DSP analysis, including region-of-interest (ROI) selection, segmentation, quality control, background assessment, and normalization protocols is given in Supplementary Data S2. Data were collected from 399 unique intraepithelial segments and 375 unique stromal segments corresponding to 184 unique tumors with either or both intraepithelial or stromal segments that passed quality control. For segment level analysis, the linear mixed model was used to calculate differential abundance of proteins. Differential expression [listed as (log) fold change (FC) in all figures and tables] was estimated fold change (FC) from the general linear model, adjusted for multiple testing (25). Correlation coefficients were determined using Spearman rank analysis of log_2_ transformed data, with Spearman *ρ* >0.5 at *P* < 0.05 considered significant.

Supplementary Figure S1 details the subsets of the TNBC cohort with PD-L1 companion assay data (whole slide or TMA) along with either RNA-seq data or high-plex DSP data.

## Results

### PD-L1 SP142 assay in whole slides

**Figure 1.**
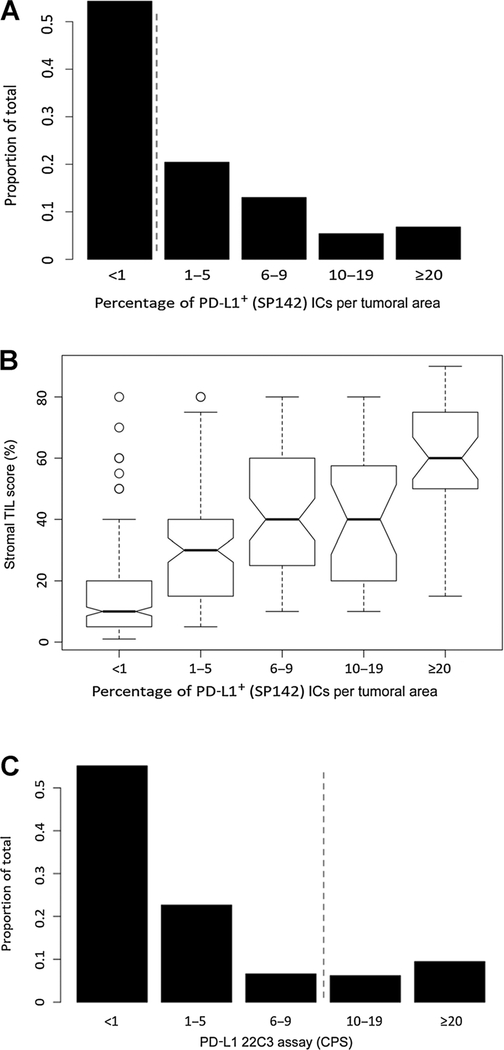
**A**, Frequency distribution of PD-L1 (SP142) whole-slide scores in TNBC cohort. Score is percentage of tumor-associated PD-L1^+^ ICs per tumoral area. Dashed line indicates cut-off point for companion diagnostic assay in TNBC (≥1% IC^+^). **B,** Binned PD-L1 (SP142) whole-slide scores as a function of stromal TIL scores. **C,** Frequency distribution of PD-L1 (22C3) CPS in TMA. CPS is defined as the number of PD-L1^+^ cells of any type (tumor cells or ICs) associated with the tumor divided by the total number of viable tumor cells. Dashed line indicates FDA-approved cut-point for companion diagnostic assay in TNBC (CPS ≥10).

From the Mayo TNBC cohort, 499 TNBC had whole slides scored with the PD-L1 SP142 assay, wherein approximately 60% of patients were postmenopausal, and most tumors were pT1-T2 (95%), pN0–1 (86%; Table 1). Using the SP142 companion assay with binary scoring (<1% IC^+^ vs. ≥1% IC^+^), 228/499 (46%) were PD-L1^+^ (IC≥ 1%), and interobserver (2-reader) overall percent agreement was 86% [kappa statistic: 0.72 (0.63–0.80)] with a positive percent agreement of 74%. Using exploratory binned scores (one reader), the majority of PD-L1^+^ TNBC (73%) were low PD-L1 expressers (<10% IC+) with heterogeneous PD-L1 staining across the tissue section (Fig. 1A). Overall, PD-L1 scores distributed as: <1% (IC+): 54%, 1–5% IC+: 21%; 6–9%: 13%; 10–19%: 5%; and ≥20%+IC: 7% (Fig. 1A). Compared with PD-L1^−^ tumors, PD-L1^+^ TNBC were associated with larger tumor size (*P* = 0.005), higher nodal stage (≥pN1: 43% vs. 31%, *P* < 0.001), higher histologic grade (grade 3: 95% vs. 86%, *P*< 0.001), and higher Ki-67 proliferation index (PI; PI>15%: 88% vs. 69%, *P* <0.001; Table 1). PD-L1^+^ TNBC were more frequently invasive ductal carcinoma (IDC) with medullary features (31% vs. 6%) compared to PD-L1− tumors. They were less frequently metaplastic (spindle or squamous)-type (4% vs. 10%, respectively) but these subtypes were infrequent overall (Table 1). Similar associations for all variables were present using an exploratory assay cut-point of ≥10% IC+. Overall, stromal TIL scores were associated with PD-L1 positivity (median score: 40% vs. 10%; *P* < 0.001), and the percentage of PD-L1+ ICs (binned groups) showed moderate positive correlation with stromal TIL scores (Spearman *r* = 0.62, *P* < 0.001; Fig. 1B). Evaluating PD-L1 expression in tumor cells, only 41 (8.2%) of TNBC had any PD-L1+ expression (all were also IC+), with the majority (35/41) demonstrating low PD-L1 expression (1%–5% of tumor cells).

### Survival analysis

The median follow-up duration for recurrence-free survival (RFS) and overall survival (OS) was 7.1 years and 7.2 years, respectively. Survival analysis was performed in the full cohort (*N* = 499), as well as patient groups that did or did not receive adjuvant chemotherapy. In the full cohort, by univariate analysis, PD-L1^+^ TNBC (SP142, ≥1% IC^+^) were associated with significantly improved RFS [HR: 0.61 (0.43–0.88), *P* = 0.007] and OS [HR: 0.73 (0.54–0.99), *P* = 0.04] compared with PD-L1^−^ tumors (Table 2; Supplementary Fig. S2). However, in a multivariate analysis including age, tumor size, tumor grade, Ki-67 PI, nodal status, stromal TIL scores, type of surgery, and receipt of adjuvant chemotherapy (yes/no), PD-L1 positivity was not independently associated with improved RFS or OS (Table 2). There was no significant association of PD-L1 tumor status with RFS or OS in the subsets of patients who did or did not receive adjuvant chemotherapy, possibly due to limited statistical power in these subgroup analyses, though PD-L1 positivity trending with better outcomes held true in all multivariate survival models.

### PD-L1 assays in TNBC cohort-derived companion TMA

**Figure 2.**
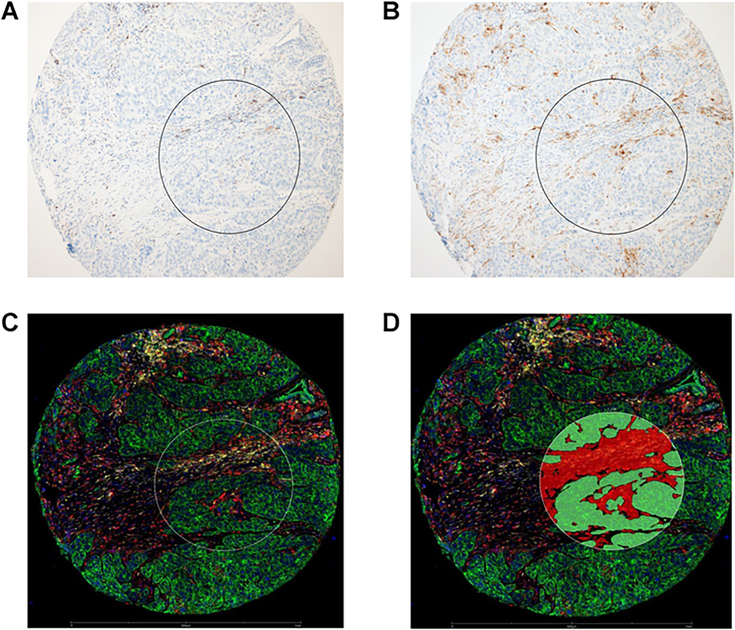
Micrographs of a representative tissue core in TNBC TMA stained with PD-L1 SP142 (**A**) or PD-L1 22C3 (**B**) antibody clones, highlighting PD-L1–positive tumor-associated ICs. **C**, Composite digital image of the same tissue core from DSP study, annotated with a 600-μm region-of-interest (white circle), and then segmented (**D**) into a cytokeratin^+^ (green fluorophore) Intraepithelial tumor segment (green overlay), and adjacent cytokeratin−/SYTO13 nuclear dye^+^ (blue fluorophore) Stroma segment (red overlay).

From the TNBC cohort of 499 tumors with whole slide PD-L1 SP142 scores, 231 were also scored in a previously constructed companion TNBC TMA (SP142 and 22C3 assays; Supplementary Fig. S1, and clinicopathologic details of the TMA in Supplementary Table S1). Overall, 99/231 (43%) of TNBC in the TMA were PD-L1^+^ (SP142), comparable with the 46% positivity rate in whole-slides (Fig. 2). The PD-L1 scores distributed as <1% IC^+^: 57%, 1%–5% IC^+^: 21%, 6%–9% IC^+^: 13%, 10%–19% IC^+^: 4%, and ≥20% IC^+^: 5%. The overall percentage agreement (OPA) between whole-slide scores and TMA scores was 81%, (kappa = 0.62, 95% CI: 0.51, 0.72). Considering the whole-slide score as the assay “gold-standard”, the false negative rate in the TMA was 19.7% (26/132) and the false positive rate was 18% (18/99). In the TMA, 86 tumors had greater than 1 scored tissue cores, wherein 10 (12%) tumors had discordant core scores (and were categorized according to the highest core score); and 8 (9% overall) had a discordant binary classification (≥1% IC+/tumoral area) that would impact the clinically-actionable cut-point of the SP142 companion assay. Similar to the whole-slide set, PD-L1 status was associated with axillary nodal status, histologic grade, and Ki-67 PI; and PD-L1 IC scores showed a moderate positive correlation with stromal TIL scores (Spearman *r* = 0.55, *P* < 0.001; Supplementary Table S1).

Overall, PD-L1 (22C3) scores in the tissue cores distributed as CPS < 1: 54%, 1–5: 24%, 6–9: 6.5%, 10–19: 6.5%, and ≥20: 9% (Figs. 1C and 2). Using the current cut-off point for the FDA-approved 22C3 companion assay (CPS ≥10), 36 of 231 (16%) of tumors were PD-L1^+^. With a cut-off point of CPS ≥1, 106/231 (46%) tumors were PD-L1+, similar to the positivity rate with the SP142 assay in the TMA (43% PD-L1^+^, SP142: ≥1% IC^+^). However, with regard to interassay agreement of the non-equivalent SP142 and 22C3 assays, the overall percent agreement with cut-points of SP142 (≥1% IC^+^) and 22C3 (CPS ≥1) was 77%, (κ = 0.54, 95% CI: 0.43–0.65) with only 76 of 129 positively-scoring tumor cores scoring PD-L1+ with both assays (positive percent agreement = 59%). The OPA decreased to 69% (kappa = 0.32, 95% CI: 0.19–0.45) when comparing the current FDA-approved cut-off points of both assays [SP142 (≥1% IC^+^) and 22C3 (CPS ≥10)], with only 32 of 103 tumor scoring PD-L1^+^ with both assays (PPA = 31%), largely due to the higher cut-off point in the 22C3 assay. Comparing cut-off points of SP142 (≥10% IC^+^) and 22C3 (CPS ≥10), the positive percent agreement was 35% largely due to lower SP142 scores in cores with CPS≥ 10. (Supplementary Table S2).

Unlike the SP142 assay, PD-L1 status with the 22C3 assay (CPS ≥1 or ≥10) was not significantly associated with histologic grade but PD-L1^+^ tumors had higher Ki-67 scores at both cut-off points (*P* < 0.002), and were more frequently IDC with medullary features (25% vs. 12% for CPS ≥1;Supplementary Table S1). Overall, stromal TIL scores were significantly higher in PD-L1^+^ tumors (median score: 40% vs. 20%; *P* < 0.001) and binned CPS scores showed moderate positive correlation with stromal TIL scores (Spearman *r* = 0.46, *P* < 0.001).

### RNA-based immune profiling of PD-L1^+^ TNBC

We applied xCell, a single-sample gene set enrichment analysis (ssGSEA) deconvolution method to obtain estimates of IC-types from bulk RNA-seq data derived from FFPE tissue sections. In the group with both RNA-seq data and whole-slide PD-L1 SP142 scores (*n* = 234, Supplementary Fig. S1), *CD274* (PD-L1) mRNA levels were positively correlated with SP142 PD-L1 scores (Spearman *r* = 0.56; *P* < 0.001). The associations of xCell features with PD-L1^+^ versus PD-L1− (SP142) TNBC categorizations are provided in Table 3. As many features had low RNA abundance, immune features with significant differences by PD-L1 status were defined as those 75th percentiles above 0.05 in the PD-L1–positive groups, and those with significant FDR *p*-values (*P* < 0.025) across comparisons of both PD-L1 cut-off points (≥1% IC^+^ and ≥10% IC^+^). Overall, as expected, PD-L1^+^ TNBC had higher immune enrichment scores than PD-L1^−^ tumors. Specifically, among lymphoid cell features, B cells, class-switched memory B cells, pro-B cells, plasma cells, CD8^+^ T cells, Th1 cells, and Th2 cells were significantly higher in PD-L1^+^ compared with PD-L1^−^ TNBC at two assay cut-off points: ≥1% IC^+^ (companion assay cut-off point) and ≥10% IC^+^ (exploratory cut-off point; *P* < 0.025; Table 3; Supplementary Table S3 for all xCell features). Among myeloid features, basophils, dendritic cells, including activated dendritic cells and plasmacytoid dendritic cells, as well as “M1-polarized” macrophages were higher in PD-L1^+^ compared with PD-L1− TNBC at both assay cut-off points (*P* < 0.025; Table 3), whereas myeloid-derived suppressor cells (MDSC) were decreased in PD-L1^+^ TNBC (*P* < 0.025).

### High-plex digital spatial immune profiling of PD-L1^+^ microenvironments

Using the Nanostring GeoMX platform, we performed spatially restricted quantitative high-plex profiling of intraepithelial tumor and adjacent stroma TIME within tumor cores to characterize and compare the immune protein milieu of PD-L1^+^ and PD-L1^−^ TNBC TIME (Fig. 2). PD-L1^+^ regions were defined as SP142 ≥1% IC^+^ (from assay data obtained on the same TMA), and PD-L1 protein levels by DSP more than 2-fold background levels (see Methods, Fig. 2, Supplementary Data S2). Among the tissue set, more than 95% of the tissue cores which were PD-L1^+^ (SP142) were also PD-L1^+^ using the 22C3 assay (CPS ≥1). Overall, there were 66 PD-L1^+^ stromal segments (and 309 PD-L1^−^ stromal segments) and 29 PD-L1^+^ intraepithelial tumor segments (and 370 PD-L1^−^ intraepithelial tumor segments), corresponding to 184 unique tumors (as some tumors had >1 tissue core in the TMA). Predictably, cytokeratin had the highest counts in intraepithelial tumor segments, and smooth muscle actin and fibronectin had the highest counts in stromal segments (Supplementary Table S4).

**Figure 3.**
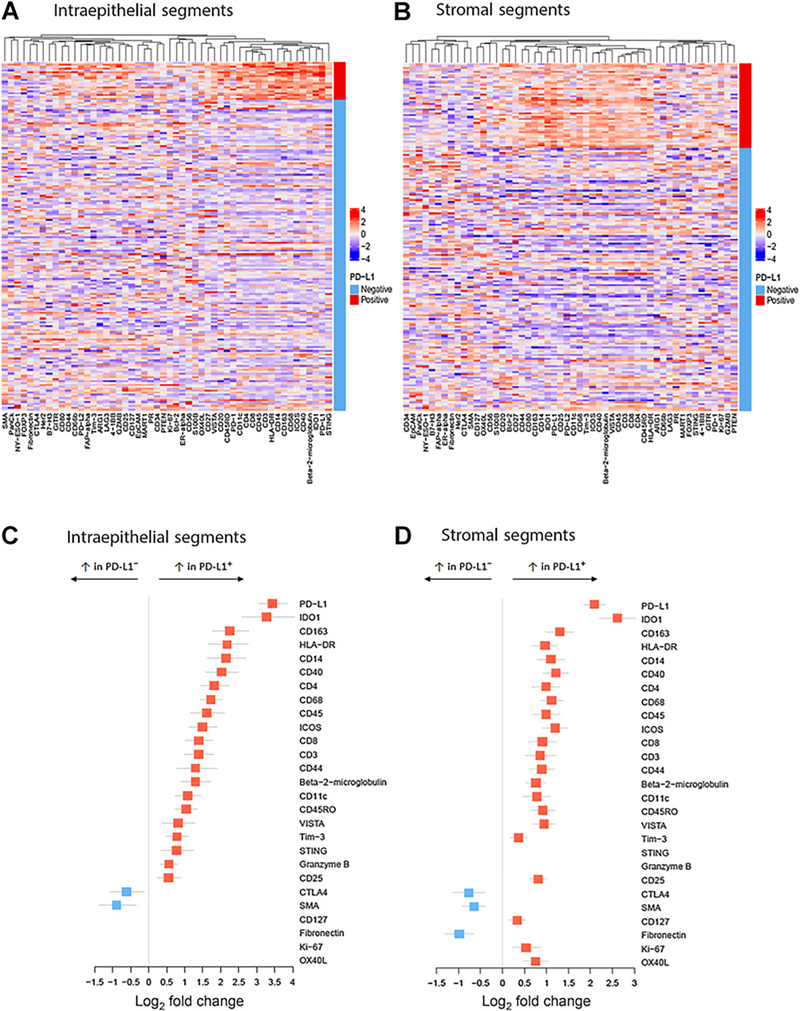
Heatmaps of high-plex DSP-derived protein abundance in intraepithelial tumor (**A**) or stromal (**B**) segments from PD-L1^+^ (red bar) versus PD-L1^−^ TNBC (light blue bar). Quantitative protein abundance data were averaged per tumor (for those with >1 tissue core in TMA), log_2_ transformed, and scaled. **C** and **D**, Forest plots of DSP data demonstrating immune protein targets with significantly increased or decreased abundance in PD-L1^+^ intraepithelial tumor segments (**C**) or stromal segments (**D**) compared with PD-L1^−^ segments (Red squares: log_2_ fold change > 0.3; adjusted *P* < 0.05; blue squares: log_2_ fold change < −0.5; adjusted *P* < 0.05).

In both segment classes, PD-L1 was a low-abundance protein, an expected finding given the low-threshold (≥1% IC^−^) for the SP142 companion assay positivity, and the observation that most PD-L1^+^ tumors are low-expressors. Compared with PD-L1^−^ segments, both the intraepithelial and stromal TIME of PD-L1^+^ segments had significantly increased counts for over 20 functionally diverse immune proteins, including myeloid and lymphoid cell-markers and multiple immune checkpoint proteins (log_2_ fold change > 0.5; adjusted *P* < 0.05; Fig. 3; Supplementary Table S4). Aside from PD-L1 protein itself, IDO-1 had the most marked increase in PD-L1^+^ segments (log_2_ FC: 2.23–3.1), followed by CD163, HLA-DR, CD14, and CD40. Among them, CD14, CD40, CD68, and CD163 protein levels had the highest (albeit moderate) positive correlations with PD-L1 protein levels (Spearman *r* ranging from 0.6–0.68 in stromal segments, *P* < 0.05).

Both macrophage markers in the panel (CD68 and CD163) were significantly higher in PD-L1+ segments (*P* < 0.001). Both CD44 and CD14 were increased in PD-L1^+^ intraepithelial and stromal segments. As these proteins are found on immune cell subsets, but also act as markers of breast cancer “stemness,” the biologic significance of this finding within the intraepithelial tumor segments may be 2-fold. The T-cell markers CD3, CD4, and CD8 were also higher in PD-L1^+^ segments, but with lower fold changes than macrophage or antigen presentation proteins (log_2_ FC range: 0.5–1, *P* < 0.025; Fig. 3). Other clinically targetable immune proteins with significant enrichment in PD-L1^+^ TIME included Stimulator of Interferon Genes (STING; a high-count protein, increased only in intraepithelial tumor segments), VISTA and Tim-3 (moderate count proteins), and OX40 L (low count protein, significantly higher only in PD-L1^+^ stromal segments; Supplementary Table S4). PD-1 and PD-L2, and 10 other proteins in the panel had insufficient protein counts above background for meaningful comparisons.

Only a few proteins were either unaltered or decreased in PD-L1^+^ segments compared with PD-L1^−^ segments. Among them, CTLA-4, smooth muscle actin, and fibronectin were significantly decreased in PD-L1^+^ stromal segments compared with PD-L1^−^ stromal segments (Fig. 3; Supplementary Table S4). B7-H3 and CD127 (IL7 receptor; both high abundance proteins) were not significantly changed between PD-L1^+^ and PD-L1^−^ intraepithelial tumor segments but showed modest changes in PD-L1^+^ stromal segments compared with PD-L1^−^ stromal segments.

## Discussion

Therapies targeting the PD-(L)1 axis have shown to improve clinical outcomes of patients with locally advanced or metastatic TNBC. Atezolizumab and pembrolizumab have different FDA-approved PD-L1 companion assays (antibody clones and scoring systems), which are not interchangeable (7). While a number of prior studies evaluated the clinicopathologic features of PD-L1^+^ TNBC, most of these studies preceded the FDA approval of specific PD-L1 companion assays for anti-PD(L)1 therapies in TNBC, and many studies focused on PD-L1 expression in tumor cells rather than ICs. In this series, we evaluated the clinicopathologic features and immune milieus of PD-L1^+^ TNBC or TIME, defined by PD-L1 companion assays. Notably, just under half of early-stage chemotherapy-naïve TNBC were PD-L1+ (SP142, whole-slide or TMA, ≥1% IC^+^), very similar to the reported frequency in a recent study of primary TNBC, or mixed primary/metastatic TNBC from the IMpassion 130 trial (1, 7, 26). While 46% of tumor cores were PD-L1^+^ with the nonclinical 22C3 cut-off point of CPS ≥1, the positive percent agreement of the two assays was only 59%. Moreover, as expected, the positive percent agreement decreased to 31% when directly comparing the FDA-approved cut-off points for the two assays (SP142: ≥ 1% IC^+^ and 22C3: CPS ≥10). While these inter-assay PD-L1 score comparisons are from a TMA and may not be representative of whole-slide scores, the data provide further support that current clinical cut-off points for PD-L1 companion assays are nonequivalent, which should be taken into consideration with selection of scoring system, interpretation, and reporting of these PD-L1 assays for clinical use.

With both companion assays, PD-L1 expression was associated with higher tumor grade and/or Ki-67 proliferation indices. With regard to histologic subtype of TNBC, PD-L1 was associated with IDC with medullary features, reflecting the enrichment of this histologic subtype with stromal TILs (9). Predictably, as PD-L1 is typically expressed in ICs rather than tumors cells in TNBC, PD-L1 positivity, with either companion assay, was significantly positively associated with stromal TILs, consistent with published data (26). In addition, we determined that the percentage of PD-L1–positive ICs (as binned scores) showed a moderate positive correlation with stromal TIL scores. These observations likely account, at least partially, for the finding that PD-L1 positivity was not independently associated with improved RFS or OS following adjustment for stromal TILs and other clinicopathologic factors.

### RNA-based immune features of PD-L1^+^ (SP142) TNBC

The modest survival benefit with anti-PD(L)-1 therapies in TNBC, seen only in a subset of patients, has prompted the search for synergistic combinatorial approaches to both augment their clinical efficacy and increase the number of patients who might benefit from immune checkpoint inhibition (27). As stromal TIL scores do not delineate specific IC populations, we characterized RNA-based immune features in PD-L1^+^ TNBC, defined by the SP142 companion assay. Deconvolution of bulk RNA-seq data demonstrated that PD-L1^+^ (SP142) tumors were associated with significant increases in immune features related to dendritic cell subsets, specifically activated dendritic cells and plasmacytoid dendritic cell, as well as M1-polarized macrophages. This supports prior observations that PD-L1 expression in tumor-associated ICs from TNBC is typically expressed by dendritic cells and macrophages, and the recent report that PD-L1 expression on macrophages was associated with higher rates of pathologic complete response to durvalumab (anti–PD-1) and chemotherapy (28). While plasmacytoid dendritic cells comprise a small, understudied fraction of tumor-infiltrating ICs, they have been implicated in breast cancer progression (29).

In addition, several T-cell subsets and B-cell subsets were increased in PD-L1^+^ (SP142) TNBC. All of these immune features also showed higher mean scores in PD-L1^+^ TNBC at an exploratory SP142 assay cut-off point of ≥10% IC^+^, suggesting there may be a “dose”-dependent relationship between the percentage of PD-L1^+^ ICs and these immune features. Notably, MDSCs were significantly lower in PD-L1^+^ tumors. MDSCs comprise a heterogeneous group of immature myeloid cells with multifaceted immunosuppressive functions, which have been implicated in breast cancer progression (30–32). Preclinical data from lung and renal cell carcinoma mouse models have demonstrated that MDSC-targeted therapy, combined with immune checkpoint enhanced antitumor activity compared with immune checkpoint inhibition alone; and early-phase clinical trials in melanoma and other tumor types are on-going (33). However, combinatorial anti-PD(L)1 and MDSC-targeted therapies remains underexplored in TNBC.

As with any bulk sequencing data, the RNA-based immune feature data serve as surrogate estimates of IC populations and are limited by the lack of spatial context – the immune features cannot be ascribed to PD-L1^+^ regions of the tumor. This limitation is particularly relevant given the low cut-off point of the SP142 companion assay (≥1% IC^+^) and the low expression of PD-L1 with heterogeneous staining in most PD-L1^+^ TNBC.

### Spatially defined immune microenvironments in PD-L1^+^ TNBC

High-plex digital profiling platforms have emerged as powerful new technologies to quantitate protein abundance with spatial context (10, 11). The Nanostring GeoMX platform has a high-plex capacity, wide dynamic range, and micron-scale spatial resolution, making it well-suited for characterization of TIME (12, 13). The recent classification of TNBC into macroscale immune phenotypes (e.g., inflamed, immune-excluded, and immune-desert tumors) underscores the potential importance of tumoral-immune environments (34). In our study, both intraepithelial and stromal PD-L1^+^ TIME were enriched in many immune proteins compared with PD-L1^−^ TIME. Overall, PD-L1^+^ TIME were most enriched in macrophage markers (CD68 and CD163), or proteins associated with antigen presentation (CD40 and HLA-DR); and PD-L1 protein levels were most highly correlated with CD68, CD163, and CD40, consistent with the observation that PD-L1 is typically expressed by antigen-presenting cells in TNBC (28). However, the DSP data offer additional insight into quantitative and spatial differences between intraepithelial and stromal PD-L1^+^ TIME. Intraepithelial tumor and adjacent stromal compartments appear to be functionally distinct TIME, and the specific localization of a subset of immune proteins, including HLA-DR, within the intraepithelial tumor compartment is associated with improved survival both in TNBC and melanoma (35, 36). In our study, a handful of proteins showed more spatially defined alterations, significantly enriched or depleted in either the PD-L1^+^ intraepithelial tumor or stromal segments. For example, fibronectin, a highly abundant protein overall, decreased significantly only in PD-L1^+^ stromal segments compared with PD-L1 stromal segments. In contrast, STING, a high abundance protein, was significantly increased only in PD-L1^+^ intraepithelial tumor segments. STING plays a critical role in IFN-dependent enhancement of antitumor immunity, and activation of STING in orthotopic models of TNBC resulted in induction of both innate and adaptive host immune responses (37).

In addition to spatial discrimination, quantitative DSP data provide insight into abundance of immune proteins in PD-L1^+^ and PD-L1^−^ TIME. Among all immune proteins increased in PD-L1^+^ TIME, IDO-1, and HLA-DR had the highest fold change with the highest absolute protein counts. IDO-1 (indoleamine 2, 3- dioxygenase) is involved in tryptophan catabolism and contributes to the both innate and adaptive immune responses. IDO-1 expression by antigen-presenting cells can induce local immune suppression, and promote systemic tolerance by activating Tregs (38). IDO-1 expression has been reported in PD-L1^+^ tumors but these studies used different cut-off points than the FDA-approved companion assays to define PD-L1 positivity (39, 40). In our study, PD-L1^+^ (SP142) TIME had significantly higher levels of IDO-1, on average 10× those of PD-L1^−^ segments, and high absolute protein counts, making it an attractive candidate protein for combinatorial targeted therapy. Clinically, the combination of IDO-1 inhibitors plus PD-(L)1 axis inhibitors have shown encouraging clinical activity in early-phase trials involving multiple tumors (not focused on TNBC), but this has not been confirmed in subsequent randomized phase III studies. For example, epacadostat plus pembrolizumab demonstrated encouraging antitumor activity in a phase I/II trial including multiple solid malignancies (only three patients with TNBC). However, the subsequent ECHO-301/KEYNOTE-252 phase III trial (focused in melanoma) did not meet its endpoint (41). While disappointing results have been seen with other combinations of IDO1 inhibitors and anti–PD(L)-1 therapies (42), these studies were generally conducted in unselected patient populations, and the clinical activity of these combinations in PD-L1- and IDO-1–expressing TNBC remains unknown.

In this study, CD40, a costimulatory protein within the tumor necrosis factor superfamily, was highly enriched in PD-L1^+^ (SP142) intraepithelial tumor and stromal segments, and may be another promising candidate for combinatorial therapies leveraging T-cell priming with checkpoint blockade (43, 44). This approach, composed of combinatorial triple therapy with a T-cell–inducing vaccine, CD40 agonist, and PD-1 antagonist antibodies effectively promoted antitumor immunity in an orthotopic breast cancer model (45).

Potential biologic and therapeutic insights may also be gleaned from CTLA-4, an immune checkpoint protein expressed on T-cell subsets, which was significantly decreased in PD-L1^+^ stromal TIME, compared with PD-L1^−^ tumors. As reported, 50%–70% of TNBC express CTLA-4 (46, 47), this observation may reflect differential CTLA-4–expressing and PD-L1–expressing TIME in TNBC, or an overall paucity of CTLA-4 in PD-L1^+^ tumors. Early clinical data of combined anti-PD-L1 (nivolumab) and anti-CTLA-4 therapy (ipilimumab) in the phase II DART trial has shown promise in a small set of patients with advanced metaplastic breast carcinoma (48). In this study, there were insufficient numbers of metaplastic carcinomas with DSP data to evaluate whether CTLA-4 abundance in PD-L1^+^ segments was associated with histologic subtypes of TNBC. This dual therapy is also being explored in HER2-negative breast cancers with high tumor mutational burden in the NIMBUS trial (NCT03789110), and the spatial localization of CTLA-4 and its functional relationship with PD-L1 in TNBC merit further investigation.

This study is not without limitations. While high-plex DSP with the Nanostring GeoMX platform can elucidate micron-scale microenvironments with quantitative protein data, neither single-cell resolution nor protein coexpression in the same cell can be determined. For example, based on our SP142 assay data and those of other studies demonstrating that PD-L1 is only rarely expressed in the neoplastic cells of TNBC, we assume that the PD-L1 protein within the intraepithelial tumor segments largely reflects expression of intercalated PD-L1^+^ ICs rather than neoplastic cells. However, we cannot ascribe the PD-L1 protein to a particular cell but rather to a micron-scale microenvironment. Similarly, plasmacytoid dendritic cells, for example, are known to coexpress IDO-1 and PD-L1, and this immune cell set may reflect our RNA-based immune feature data and DSP data in PD-L1^+^ (SP142) TIME; however, additional study is required to test this hypothesis. Nevertheless, the high sensitivity and wide dynamic range of this platform offers heretofore unseen insights into the protein-based immune architecture of PD-L1^+^ TIME, and to the relative abundance of these proteins. While ideally it would have been informative to compare PD-L1^+^ and PD-L1^−^ tissue cores from the same tumors, there was an insufficient number of tumors in the TMA with multiple tissue cores per tumor and discordant PD-L1 scores in those cores for meaningful analysis.

In summary, approximately half of early-stage, chemotherapy-naïve TNBC were PD-L1^+^ using the SP142 companion assay (≥1% IC^+^). In contrast, 16% of tumors in the TMA were PD-L1^+^ with the 22C3 assay using the FDA-approved cut-off point of CPS≥10%. Most PD-L1^+^ TNBC were low PD-L1 expressors with heterogeneous staining and were higher grade tumors with higher TILs. By RNA profiling, PD-L1^+^ (SP142) TNBC had increased expression of genes associated with dendritic cell subsets, macrophages, and T/B lymphocyte subsets, with decreased MDSCs. PD-L1^+^ (SP142) intraepithelial tumor and stroma TIME were similar but not identical, highly enriched in IDO-1, HLA-DR, CD163, and CD40 compared with PD-L1− TIME, but with segment-specific alterations in functionally diverse immune proteins (i.e., CTLA-4, STING, fibronectin). These data lend insight into the functional immune microenvironments of PD-L1^+^ TNBC and may inform future studies on combinatorial approaches to PD-(L)1 axis therapies for this patient population.

## Supplementary Material

Supplementary Table 4

Supplementary Table 3

Supplemental Figure 2

Supplementary Data 1

Supplementary Data 2

Supplementary Table S2

Supplemental Figure 1

Supplementary Table S1

## Figures and Tables

**Table 1.** Clinicopathologic features of PD-L1^+^ and PD-L1^−^ (SP142) TNBC (whole-slide scores).

	PD-L1 SP142 assay* (whole-slide)		
	%IC+ <1 (*N* = 271)	%IC+ ≥1 (*N* = 271)	*P* value
Menopausal status			0.36
Postmenopausal	169 (62.4%)	133 (58.3%)	
Pre/perimenopausal	102 (37.6%)	95 (41.7%)	
Age (y)			0.72
≥50	180 (66.4%)	148 (64.9%)	
<50	91 (33.6%)	80 (35.1%)	
Tumor size			0.005
Missing	0	1	
≤2.0 cm	154 (56.8%)	98 (43.2%)	
2.1–5.0 cm	102 (37.6%)	118 (52.0%)	
≥5.1 cm	15 (5.5%)	11 (4.8%)	
Axillary nodal status			0.0003
Missing	6	1	
0	184 (69.4%)	128 (56.4%)	
1–3+	60 (22.6%)	56 (24.7%)	
4–9+	15 (5.7%)	19 (8.4%)	
≥10+	6 (2.3%)	24 (10.6%)	
Nottingham grade			0.0006
Grade 1–2	38 (14.0%)	11 (4.8%)	
Grade 3	233 (86.0%)	217 (95.2%)	
Ki-67 Proliferation index			<1e-04
Missing	2	0	
≤15%	82 (30.5%)	28 (12.3%)	
15.1%–30%	54 (20.1%)	48 (21.1%)	
≥30%	133 (49.4%)	152 (66.7%)	
Stroma TIL scores (%)			<1e-04
Median (range)	10.0 (1.0–80.0)	40.0 (5.0–90.0)	
Histologic subtypes			<1e-04
Apocrine	21 (7.7%)	5 (2.2%)	
Invasive carcinoma NST	206 (76.0%)	143 (62.7%)	
Medullary	16 (5.9%)	71 (31.1%)	
Metaplastic	28 (10.3%)	9 (3.9%)	
Surgery			0.06
Lumpectomy	143 (52.8%)	101 (44.3%)	
Mastectomy	128 (47.2%)	127 (55.7%)	
Adjuvant radiation			0.82
Missing	35	51	
No	104 (44.1%)	80 (45.2%)	
Yes	132 (55.9%)	97 (54.8%)	
Adjuvant chemotherapy			0.002
Missing	33	49	
No	97 (40.8%)	47 (26.3%)	
Yes	141 (59.2%)	132 (73.7%)	

**Table 2.** Survival analysis in PD-L1^+^ TNBC versus PD-L1^−^ TNBC (SP142 assay, whole-slide scores).

**Univariate** **PD-L1^+^ TNBC (SP142)**	**RFS**		**OS**	
	**HR (95% CI)**	***P***	**HR (95% CI)**	***P***

Entire cohort	0.61 (0.43–0.88)	0.01	0.73 (0.54–0.99)	0.04
Without chemotherapy	0.61 (0.32–1.16)	0.13	0.66 (0.39–1.10)	0.11
With chemotherapy	0.73 (0.47–1.15)	0.17	0.74 (0.47–1.16)	0.19

**Multivariate** **PD-L1^+^ TNBC (SP142)**	**RFS**		**OS**	
	**HR (95% CI)**	***P***	**HR (95% CI)**	***P***

Entire cohort	0.66 (0.415–1.06)	0.09	0.82 (0.54–1.27)	0.38
With chemotherapy	0.70 (0.40–1.23)	0.22	0.70 (0.40–1.22)	0.21
Without chemotherapy	0.49 (0.20–1.22)	0.12	0.75 (0.34–1.67)	0.48

* SP142 assays scored as % tumor-associated immune cells/tumor area (see Methods).

**Table 3.** Gene set-associated immune cell estimated proportions by PD-L1 status.

	PD-L1 status by whole-slide SP142 assay			
	Companion assay cut-off point (≥1% ICs+)		Exploratory cut-off point (≥10% ICs+)	
Immune feature	PD-L1^−^ (*N* = 118)	PD-L1^+^ (*N* = 116)	PD-L1^−^ (*N* = 199)	PD-L1^+^ (*N* = 35)
B cells	0.00 (0.00–0.01)	0.04* (0.00–0.16)	0.00 (0.00–0.04)	0.12* (0.02–0.21)
Basophils	0.04 (0.00–0.09)	0.07* (0.03–0.14)	0.05 (0.00–0.11)	0.12* (0.04–0.20)
Class switched memory B cells	0.01 (0.00–0.03)	0.03* (0.01–0.07)	0.01 (0.00–0.04)	0.05* (0.02–0.08)
Pro-B cells	0.00 (0.00–0.02)	0.03* (0.00–0.06)	0.00 (0.00–0.03)	0.06* (0.03–0.09)
Plasma cells	0.01 (0.00–0.02)	0.03* (0.01–0.05)	0.02 (0.01–0.04)	0.03* (0.02–0.07)
CD8^+^ T Cells	0.00 (0.00–0.02)	0.03* (0.00–0.07)	0.00 (0.00–0.03)	0.05* (0.02–0.13)
Th1 cells	0.01 (0.00–0.05)	0.05* (0.00–0.08)	0.02 (0.00–0.06)	0.07* (0.01–0.10)
Th2 cells	0.14 (0.08–0.20)	0.17* (0.12–0.23)	0.15 (0.10–0.20)	0.21* (0.14–0.26)
Dendritic cells	0.01 (0.00–0.04)	0.04* (0.03–0.06)	0.03 (0.01–0.05)	0.05* (0.03–0.06)
aDC	0.29 (0.18–0.40)	0.49* (0.37–0.61)	0.35 (0.25–0.51)	0.57* (0.44–0.65)
cDC	0.08 (0.05–0.16)	0.13* (0.08–0.22)	0.10 (0.05–0.17)	0.13 (0.05–0.26)
iDC	0.21 (0.06–0.35)	0.25 (0.07–0.42)	0.25 (0.08–0.40)	0.17 (0.02–0.36)
pDC	0.01 (0.00–0.03)	0.07* (0.02–0.15)	0.02 (0.00–0.06)	0.14* (0.08–0.20)
Macrophages	0.01 (0.00–0.04)	0.03* (0.01–0.06)	0.02 (0.00–0.05)	0.04* (0.02–0.07)
M1 subtype	0.01 (0.00–0.03)	0.04* (0.02–0.06)	0.02 (0.01–0.04)	0.04* (0.03–0.06)
M2 subtype	0.03 (0.01–0.04)	0.03 (0.01–0.04)	0.03 (0.01–0.04)	0.02 (0.01–0.05)
MDSC	0.38 (0.24–0.52)	0.21* (0.10–0.36)	0.32 (0.19–0.48)	0.14* (0.02–0.32)

Note: Representative data from the X cell tool. Summary statistics are Median (Q1, Q3) wherein * indicates a statistically-significant comparison (with *P* values from Kruskal–Wallis rank-based test, adjusted by FDR where adjusted *P* values <0.025) as higher or lower in PD-L1^+^ group by either cut-off point.

Abbreviations: aDC, activated dendritic cells; cDC, conventional dendritic cells; iDC, immature dendritic cells; pDC, plasmacytoid dendritic cells. Refer to manuscript for full names of features (https://genomebiology.biomedcentral.com/articles/10.1186/s13059-017-1349-1).

## Translational Relevance

Programmed death ligand 1 [PD-(L)1]-targeted immunotherapies have shown modest survival benefit in advanced triple-negative breast cancer (TNBC) and improved pathologic response in the neoadjuvant setting. Using the FDA-approved PD-L1 SP142 and 22C3 companion assays, we identified clinicopathologic characteristics, RNA-based immune features, and spatially constrained protein-based tumor–immune microenvironments (TIME) of early-stage PD-L1^+^ TNBC. Forty-six percent of TNBC were PD-L1^+^ with the SP142 assay, and 16% were PD-L1^+^ with the 22C3 assay. PD-L1^+^ TNBC were higher grade and had higher tumor-infiltrating lymphocytes (TIL). With deconvolution of RNA data, PD-L1^+^ TNBC had increased dendritic cell, macrophage, and lymphocytic immune features, with decreased myeloid-derived suppressor cells. By high-plex digital spatial profiling, PD-L1^+^ stroma and intraepithelial TIME were highly enriched in IDO-1, HLA-DR, CD40, and CD163, and had TIME-specific alterations in functionally diverse immune proteins [CTLA-4, Stimulator of Interferon Genes (STING), fibronectin]. These data provide biologic insight and identify candidate targets for future studies of combinatorial immunotherapies in patients with PD-L1^+^ TNBC.
