# Supplementary material for: Characteristics and Spatially Defined Immune (micro)landscapes of Early-stage PD-L1–positive Triple-negative Breast Cancer
Source: Clin Cancer Res. Author manuscript; Available in PMC 2022 Feb 2. (PMC8808363; doi:10.1158/1078-0432.CCR-21-0343)
Supplement: Supplementary Table 4 [file NIHMS1767618-supplement-Supplementary_Table_4.docx]

**Supplementary Table S4: Abundance of Immune Proteins and other Biomarkers in PD-L1+ segments vs. PD-L1- Segments using high-plex digital spatial profiling**

|  | **Intraepithelial Tumor Segments** | | | | **Stromal Segments** | | | |
| --- | --- | --- | --- | --- | --- | --- | --- | --- |
| **Protein** | **log2FC (CI)**  **PD-L1+ vs. PD-L1-** | **adjusted p value*** | **PD-L1+ Segments**  **Mean Counts**  **(N=29)** | **PD-L1- Segments**  **Mean Counts**  **(N=370)** | **log2FC (CI)**  **PD-L1+ vs. PD-L1-** | **adjusted p value*** | **PD-L1+ Segments**  **Mean Counts**  **(N=66)** | **PD-L1- Segments**  **Mean Counts**  **(N=309)** |
| PD-L1 | **3.44 (3.05, 3.86)** | **< 0.001** | 217.6 | 19.6 | **2.09 (1.84, 2.35)** | **< 0.001** | 95.4 | 20.5 |
| IDO1 | **3.27 (2.59, 4.05)** | **< 0.001** | 1760.9 | 175.5 | **2.61 (2.2, 3.04** | **< 0.001** | 675.0 | 99.0 |
| CD163 | **2.25 (1.77, 2.77)** | **< 0.001** | 339.5 | 77.6 | **1.3 (0.98, 1.63)** | **< 0.001** | 502.9 | 218.4 |
| HLA-DR | **2.18 (1.65, 2.76)** | **< 0.001** | 3158.6 | 662.5 | **0.97 (0.67, 1.27)** | **< 0.001** | 1576.0 | 734.8 |
| CD14 | **2.15 (1.64, 2.7)** | **< 0.001** | 710.7 | 196.5 | **1.1 (0.8, 1.42)** | **< 0.001** | 474.0 | 223.5 |
| CD40 | **2.02 (1.59, 2.49)** | **< 0.001** | 263.3 | 61.5 | **1.21 (0.93, 1.5)** | **< 0.001** | 240.4 | 96.1 |
| CD4 | **1.82 (1.44, 2.23)** | **< 0.001** | 802.7 | 220.2 | **0.99 (0.69,1.3)** | **< 0.001** | 994.8 | 472.5 |
| CD68 | **1.73 (1.43, 2.04)** | **< 0.001** | 1293.3 | 392.1 | **1.12 (0.86,1.38)** | **< 0.001** | 1594.0 | 679.4 |
| CD45 | **1.61 (1.17, 2.1)** | **< 0.001** | 1755.9 | 558.5 | **0.99 (0.68,1.31)** | **< 0.001** | 2621.6 | 1224.9 |
| ICOS | **1.5 (1.13, 1.89)** | **< 0.001** | 120.3 | 42.7 | **1.19 (0.9,1.49)** | **< 0.001** | 128.1 | 51.9 |
| CD3 | **1.39 (1.01, 1.79)** | **< 0.001** | 514.3 | 188.7 | **0.86 (0.52,1.21)** | **< 0.001** | 843.6 | 420.4 |
| CD8 | **1.39 (1.02, 1.79)** | **< 0.001** | 565.4 | 213.0 | **0.91 (0.59,1.24)** | **< 0.001** | 554.4 | 284.7 |
| Beta-2-microglobulin | **1.3 (0.9, 1.73)** | **< 0.001** | 2840.4 | 1122.2 | **0.76 (0.54,0.99)** | **< 0.001** | 1761.0 | 973.0 |
| CD44 | **1.3 (0.75, 1.9)** | **< 0.001** | 6750.3 | 2653.1 | **0.89 (0.6,1.19)** | **< 0.001** | 3637.1 | 1762.8 |
| CD11c | **1.08 (0.73,1.46)** | **< 0.001** | 554.3 | 241.1 | **0.78 (0.48,1.08)** | **< 0.001** | 882.8 | 457.8 |
| CD45RO | **1.03 (0.73,1.35)** | **< 0.001** | 131.1 | 65.5 | **0.92 (0.65,1.19)** | **< 0.001** | 152.3 | 76.5 |
| VISTA | **0.81 (0.37,1.28)** | **< 0.001** | 183.9 | 95.3 | **0.95 (0.7,1.2)** | **< 0.001** | 252.4 | 121.0 |
| Tim-3 | **0.78 (0.48,1.11)** | **< 0.001** | 264.9 | 135.2 | 0.37 (0.17,0.56) | **< 0.001** | 207.9 | 138.2 |
| Granzyme B | **0.56 (0.33,0.8)** | **< 0.001** | 302.9 | 209.7 | 0.16 (-0.11,0.43) | 1 | 203.8 | 171.6 |
| STING | **0.77 (0.34,1.24)** | **0.0093** | 1490.9 | 747.7 | 0.24 (-0.08,0.58) | 0.89 | 1519.4 | 1004.3 |
| CD25 | **0.55 (0.25,0.88)** | **0.0093** | 100.2 | 66.4 | **0.81 (0.62,1.01)** | **< 0.001** | 76.0 | 40.4 |
| Smooth muscle actin | **-0.89 (-1.38,-0.35)** | **0.0093** | 6215.4 | 10680.1 | **-0.64(-0.9,-0.37)** | **< 0.001** | 11977.7 | 17166.2 |
| CTLA4 | -0.62 (-1.08,-0.13) | 0.076 | 97.2 | 138.6 | **-0.77(-1.13,-0.39)** | **< 0.001** | 89.0 | 128.0 |
| CD56 | -0.48 (-0.86,-0.06) | 0.15 | 100.1 | 134.2 | 0.11(-0.06,0.27) | 1 | 72.1 | 70.2 |
| OX40L | 0.38 (-0.05,0.83) | 0.65 | 132.6 | 98.9 | **0.75(0.47,1.03)** | **< 0.001** | 98.8 | 53.9 |
| Ki-67 | 0.25(-0.21,0.75) | 1 | 906.7 | 693.6 | **0.54(0.22,0.86)** | **0.008** | 280.3 | 184.5 |
| Histone H3 |  |  | 25217.3 | 26711.2 | 0.3(0.1,0.5) | 0.02 | 14972.9 | 12500.5 |
| Pan-cytokeratin | -0.16(-0.65,0.38) | 1 | 6409.0 | 7036.6 | -0.72(-1.09,-0.33) | **< 0.001** | 218.2 | 374.0 |
| Fibronectin | 0.06(-0.33,0.48) | 1 | 1449.0 | 1359.9 | **-0.98(-1.3,-0.65)** | **< 0.001** | 2534.3 | 4951.1 |
| CD127 | 0.19(-0.12,0.52) | 1 | 1110.2 | 934.8 | 0.33(0.15,0.52) | < 0.001 | 405.1 | 314.5 |
| B7-H3 | 0.21 0.23,0.69) | 1 | 2080.8 | 1584.1 | -0.41(-0.67,-0.14) | 0.02 | 1103.3 | 1280.0 |
| CD34 | 0.06(-0.24,0.38) | 1 | 213.1 | 254.6 | -0.39(-0.68,-0.09) | 0.06 | 425.9 | 637.3 |
| ARG1** | - | - | 52.7 | 38.7 |  |  |  |  |
| CD20** | - | - | 73.7 | 52.7 |  |  |  |  |
| CD27** | - | - | 18.9 | 53.0 |  |  |  |  |
| CD66b** | - | - | 25.0 | 18.3 |  |  |  |  |
| CD80** | - | - | 24.8 | 10.8 |  |  |  |  |
| FAP alpha** | - | - | 63.6 | 53.9 |  |  |  |  |
| FOXP3** | - | - | 26.2 | 24.8 |  |  |  |  |
| GITR** | - | - | 39.4 | 14.9 |  |  |  |  |
| LAG-3** | - | - | 21.7 | 14.9 |  |  |  |  |
| PD-1** | - | - | 29.9 | 23.7 |  |  |  |  |
| PD-L2** | - | - | 29.4 | 18.9 |  |  |  |  |
| X4-1BB** | - | - | 30.0 | 19 |  |  |  |  |

*Bolded: log 2FC> 0.5; with adjusted p value < 0.05; bolded in red log 2FC< - 0.5; with adjusted p value < 0.05

**Insufficient counts above background, protein excluded from analysis
