# Supplementary material for: Characteristics and Spatially Defined Immune (micro)landscapes of Early-stage PD-L1–positive Triple-negative Breast Cancer
Source: Clin Cancer Res. Author manuscript; Available in PMC 2022 Feb 2. (PMC8808363; doi:10.1158/1078-0432.CCR-21-0343)
Supplement: Supplementary Data 2 [file NIHMS1767618-supplement-Supplementary_Data_2.docx]

**Supplementary Data S1: GeoMX Digital Spatial Profiling Expanded Protocol.**

NanoString GeoMX digital spatial profiling (DSP) was used to quantify antibody binding to user-defined regions of interest (ROIs) in 5-micron sections obtained from a previously-constructed TNBC cohort-derived companion TMA. The TMA is composed of 1-2 x 1 mm formalin-fixed, paraffin-embedded (FFPE) TNBC tumor cores obtained from representative tumor blocks (See Methods). Slides were stained using conventional immunohistochemistry technology, as described in the GeoMX protocol (I).

**Antibody staining:** All slides were stained with an antibody cocktail that contained three fluorophore-labeled “morphological” antibodies and SYTO-13 nuclear dye, used to guide ROI selection and masking. Anti-pan-cytokeratin (Alexafluor 532), anti-CD45 (Alexafluor 594), and the nuclear stain SYTO13 were obtained from NanoString and were incubated with slides at a 1:40 dilution. Anti-CD68 (Alexafluor 647) was purchased from Novus Biologicals and used at a concentration of 0.25 µg/mL. Of note, for this experiment, neither the CD45 or CD68 staining patterns were used for ROI selection (see below). In addition to the “morphological” cocktail, the slides were incubated with a commercially-available antibody cocktail (Nanostring) composed of 52 target antibodies plus 6 control antibodies, each linked to an unique oligodeoxynucleotide “bar code” by a UV-labile cross linker (listed in Appendix). Each of these oligodeoxynucleotide-conjugated antibodies had been validated by the vendor using a rigorous, multistep process in both FFPE tissues and cell pellets to ensure optimal sensitivity, specificity and reproducibility for high-plex antibody panels for the analysis of FFPE sections (II-IV).

**Image capture:** Each TMA slide (N= 5 slides due to large size of TMA) was scanned on the GeoMX digital analyzer and a composite four-color/channel digital image was generated based on the three-fluorophore-tagged antibodies and SYTO-13 nuclear dye. Due to limitations on the size of the GeoMX scanning area, we obtained data only on a subset of the TNBC in the TMA (N= 184 unique tumors).

**ROI selection:** An immunofluorescent image from a representative core is shown in Figure panel A. Per tissue core, a 600-micron circular ROI was selected by an experienced breast pathologist (Dr. Carter) to capture a region enriched in viable tumor with adjacent stroma (Figure B). This strategy was devised to maximize signal from antibodies bound to intraepithelial (cytokeratin-positive) and stromal (SYTO13-positive/cytokeratin-negative) regions. ROI selection was blinded to PD-L1 SP142 or 22C3 immunohistochemical assay status; however, for assignment of PD-L1+ or PD-L1- status for this study, PD-L1 immunohistochemical assay status was visually confirmed by the study pathologist after the DSP experiment was complete.

**ROI segmentation:** Segmentation was carried out by an experienced technologist (J.M. Kachergus). ROI segmentation involved assignment of “masks” to encompass the **intraepithelial segment** composed of the cytokeratin-positive cells (Figure panel C, light green mask) and the **stromal segment** composed of SYTO13-positive/cytokeratin-negative cells (Figure panel D, dark red mask) segments. Each ROI was inspected visually to ascertain that the computer-generated masks precisely encompassed the appropriate segments. These masks define, within each ROI, areas of illumination (AOIs) or segments which guide the computer-driven UV laser. The laser illuminates each masked-segment type sequentially to liberate the antibody-bound oligonucleotides. Intraepithelial (cytokeratin-positive) segments were illuminated first, followed by stromal segments (cytokeratin-negative/SYTO13-positive). The reproducibility of the masking process has been previously documented (II-IV) and was also verified by manually reviewing the masks and comparing the amount of cytokeratin protein found in the intraepithelial and stromal segments (with the intraepithelial segments having, as expected, an order of magnitude more cytokeratin protein than the stromal segments). The liberated oligonucleotides were collected in an automated fashion and deposited in a 96-well plate. Released oligonucleotides were quantitated using conventional NanoString nCounter technology with a GeoMX codeset (II-IV).

**Quality control and background assessment:** Raw counts, corresponding to the number of oligonucleotides per segment, were exported to the GeoMX software package for quality control and data normalization protocols. The GeoMX software captures the number of SYTO13-positive nuclei in each segment, as well as the surface area of each segment. Segments with fewer than 20 nuclei or a surface area of <1600 squared microns were filtered from further analysis, as recommended by the NanoString user’s manual (I). The segments that remained were then scaled using a positive hybridization control that corrects for variation during nCounter analysis. Background (presumptive non-specific) binding of antibodies was assessed using three negative control antibodies: Mouse (Ms) IgG1, Rabbit (Rb) IgG, and Mouse (Ms) IgG2a. Analysis of counts from the negative controls revealed that MsIgG1 and RbIgG correlated well, whereas counts from MsIgG2a did not correlate with either of the other two negative controls. Consequently, the geometric means of MsIgG1 and RbIgG were used to define background levels.

Background subtraction was not carried out as it can overestimate fold change in low abundance proteins. Background for each segment was defined as 2X the geomean of the two negative controls (i.e. background was determined separately for each AOI for intraepithelial and stromal segments). Probes with background or lower counts in >90% of segments were flagged. These probes were not filtered because, in some cases, the 10% or so of tumors that express higher than background counts might have biological significance (e.g. these samples might identify a set of TNBC tumors with discrete clinical or pathological features of interest).

**Data normalization:** The NanoString GeoMX antibody cocktail reagents include antibodies to three housekeeping proteins: Histone H3, ribosomal protein S6, and GAPDH. In our dataset, Histone H3 and S6 correlated well, whereas GAPDH did not correlate with either of the other housekeeping proteins, exhibiting a lower correlation coefficient and a different slope of the linear regression line describing GAPDH vs Histone H3 or S6, compared to Histone H3 vs S6. Consequently, normalization was carried out using the geomean of Histone H3 and S6.

Each protein within a given segment was first divided by the geomean of Histone H3 and S6 within that segment. The resultant values were then multiplied by the average geomean of Histone H3 and S6 for all segments within that category. In parallel, we used signal to noise ratio (SNR) for normalization. SNR is defined as the counts measured for each protein in a given segment (signal) divided by the geomean of MsIgG1 and RbIgG (negative controls, noise) in that segment. In general, the results obtained with SNR-based data normalization were comparable to those derived from housekeeping protein normalization. However, SNR analysis is biased by the assumption that noise represents non-specific antibody binding, which is assumed to vary as a direct function of total cellular content in the segment. In fact, a significant subset of segments (up to 20%) have very low noise (geomean negative controls <20 counts), and noise in these samples does not correlate with number of nuclei in the segment and is therefore not attributable to non-specific antibody binding. Consequently, one ends up normalizing such samples to random machine noise, rather than non-specific binding. Rather than filter these low noise segments, we used housekeeping protein-based data normalization, rather than signal to noise ratio. Personal communications with NanoString and other experienced GeoMX users indicates that, at this time, housekeeping-based data normalization is the accepted standard protocol.


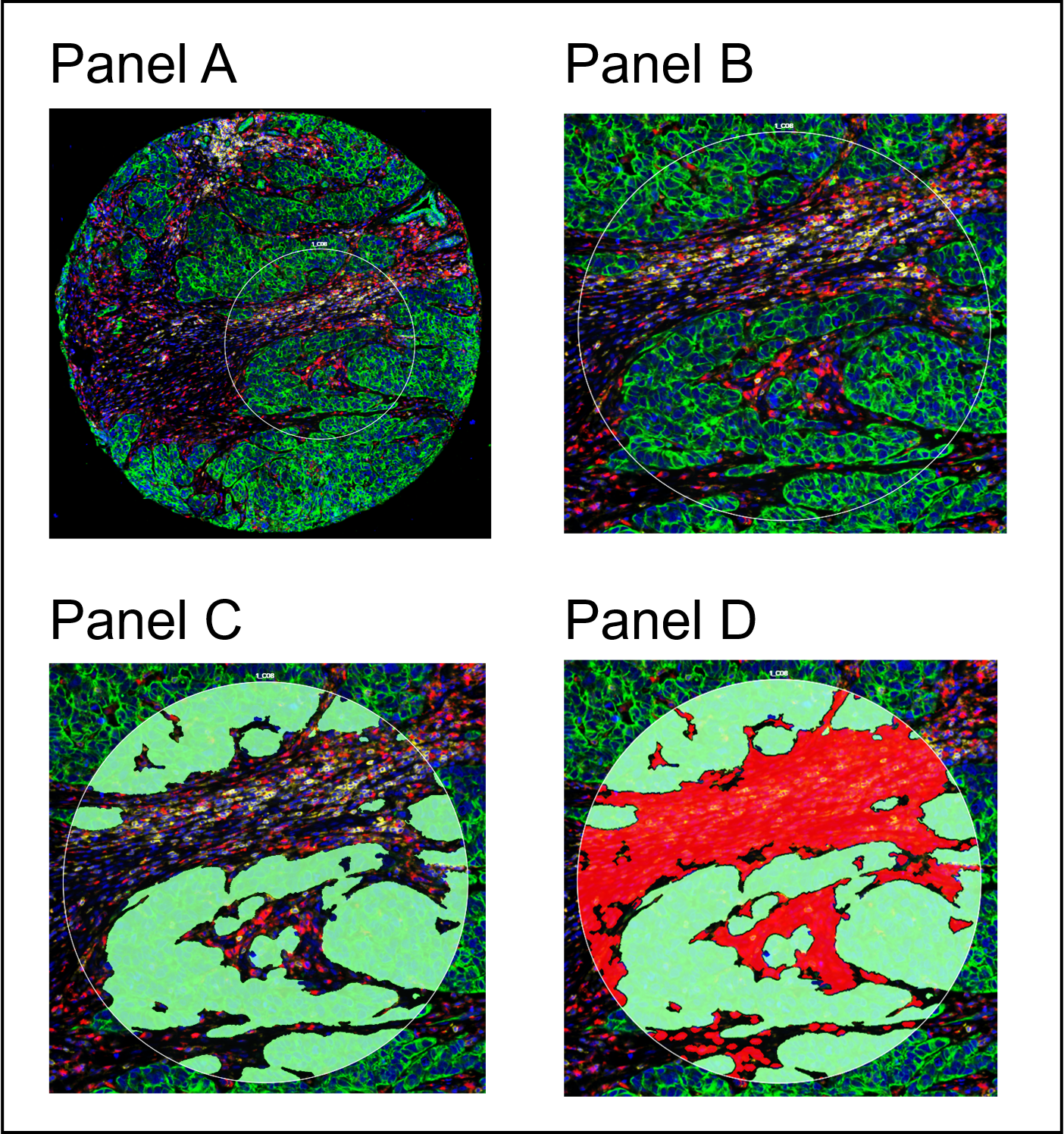


**Figure 1: Segmentation of ROIs.** **Panel A** is a composite digital image of an immunostained tissue core from the TNBC TMA. Green is cytokeratin-positive epithelial (tumor) cells. Yellow is CD45-positive leukocytes and red is CD68-positive macrophages. Nuclei are stained blue with SYTO-13 dye. **Panel B** shows a 600-micron ROI, which is segmented to generate an intraepithelial mask (light green, **Panel C**) that is restricted to the cytokeratin-positive cells with the ROI whereas the (adjacent) stromal mask (red, **Panel D**), encompasses the cytokeratin-negative/SYTO13-positive cells within the ROI.

Relevant user’s manuals and white papers

1. NanoString GeoMX user’s manual <https://blog.nanostring.com/geomx-online-user-manual/Content/Home2.0.htm>)
2. BioLegend Tech Note: Simultaneous profiling of protein and RNA with 3D Biology technology: Assay validation and immuno-oncology application.
3. Hinerfeld et al., 2019 *Validation of Antibody Panels for High-Plex Immunohistochemistry Applications* J Biomol Tech 30(suppl): S40-S41.
4. Merritt CR, Ong GT, Church SE, Barker K, Danaher P, Geiss G, Hoang M, Jung J, Liang Y, McKay-Fleisch J, Nguyen K, Norgaard Z, Sorg K, Sprague I, Warren C, Warren S, Webster PJ, Zhou Z, Zollinger DR, Dunaway DL, Mills GB, Beechem JM. Multiplex digital spatial profiling of proteins and RNA in fixed tissue. Nat Biotechnol. 2020 May;38(5):586-599. doi: 10.1038/s41587-020-0472-9. Epub 2020 May 11. PMID: 32393914.

Appendix: List of oligodeoxynucleotide-conjugated antibodies in high-plex cocktail

|  |
| --- |
| \| **Antibody Target Name** \| \| \| --- \| --- \| \| ARG1 \|  \| \| B7-H3 \|  \| \| Bcl-2 \|  \| \| Beta-2-microglobulin \|  \| \| CD11c \|  \| \| CD127 \|  \| \| CD14 \|  \| \| CD163 \|  \| \| CD20 \|  \| \| CD25 \|  \| \| CD27 \|  \| \| CD3 \|  \| \| CD34 \|  \| \| CD4 \|  \| \| CD40 \|  \| \| CD44 \|  \| \| CD45 \|  \| \| CD45RO \|  \| \| CD56 \|  \| \| CD66b \|  \| \| CD68 \|  \| \| CD8 \|  \| \| CD80 \|  \| \| CTLA4 \|  \| \| EpCAM \|  \| \| Estrogen receptor alpha \|  \| \| FAP alpha \|  \| \| Fibronectin \|  \| \| FOXP3 \|  \| \| GAPDH \| Housekeeping protein \| \| GITR \|  \| \| Granzyme B \|  \| \| HER2 \|  \| \| Histone-H3 \| Housekeeping protein \| \| HLA-DR \|  \| \| ICOS \|  \| \| IDO1 \|  \| \| Ki-67 \|  \| \| LAG3 \|  \| \| MART1 \|  \| \| MsIgG1 \| Negative Control \| \| MsIgG2a \| Negative Control \| \| NY-ESO-1 \|  \| \| OX40L \|  \| \| Pan-cytokeratin \|  \| \| PD-L1 \|  \| \| PD-1 \|  \| \| PD-L2 \|  \| \| Progesterone receptor \|  \| \| PTEN \|  \| \| Rb IgG \| Negative Control \| \| S100B \|  \| \| S6 (Ribosomal) \| Housekeeping protein \| \| Smooth muscle actin \|  \| \| STING \|  \| \| Tim-3 \|  \| \| VISTA \|  \| \| X4.1BB \|  \| |
|  |
|  |
|  |
|  |
|  |
|  |
|  |
|  |
|  |
|  |
|  |
|  |
|  |
|  |
|  |
|  |
|  |
|  |
|  |
|  |
|  |
|  |
|  |
|  |
|  |
|  |
|  |
|  |
|  |
|  |
|  |
|  |
|  |
|  |
|  |
|  |
|  |
|  |
|  |
|  |
|  |
|  |
|  |
|  |
|  |
|  |
|  |
|  |
|  |
|  |
|  |
|  |
|  |
|  |
|  |
|  |
|  |
