# Supplementary material for: Characteristics and Spatially Defined Immune (micro)landscapes of Early-stage PD-L1–positive Triple-negative Breast Cancer
Source: Clin Cancer Res. Author manuscript; Available in PMC 2022 Feb 2. (PMC8808363; doi:10.1158/1078-0432.CCR-21-0343)
Supplement: Supplementary Table S2 [file NIHMS1767618-supplement-Supplementary_Table_S2.docx]

**Supplementary Table S2: Inter-Assay Agreement between PD-L1 SP142 and 22C3 Assay Scores in TNBC TMA**

| SP142  Score  (%IC+)* | 22C3  Score  (CPS)** | | Total | Positive  Percent  Agreement | Negative  Percent  Agreement | Overall  Percent  Agreement | Kappa Statistic (95% CI) |
| --- | --- | --- | --- | --- | --- | --- | --- |
|  | **<1** | **≥1** |  |  |  |  |  |
| **<1** | 102 | 30 | 132 | 59% | 66% | 77% | 0.54  (0.43, 0.65) |
| **≥1** | 23 | 76 | 99 |  |  |  |  |
|  | 125 | 106 | **231** |  |  |  |  |
|  | | | | | | | |
|  | **<10** | **≥10** |  | | | | |
| **<10** | 188 | 21 | 209 | 35% | 87% | 88% | 0.45  (0.26, 0.64) |
| **≥10** | 7 | 15 | 22 |  |  |  |  |
|  | 195 | 36 | **231** |  |  |  |  |
|  | | | | | | | |
|  | **< 10** | **≥10** |  | | | | |
| **< 1** | 128 | 4 | 132 | 31% | 64% | 69% | 0.32  (0.19, 0.45) |
| **≥1** | 67 | 32 | 99 |  |  |  |  |
|  | 195 | 36 | **231** |  |  |  |  |

*SP142 assays scored as % tumor-associated immune cells/tumor area; **22C3 assay scored as combined positive score (see Methods).
