# Supplementary material for: Characteristics and Spatially Defined Immune (micro)landscapes of Early-stage PD-L1–positive Triple-negative Breast Cancer
Source: Clin Cancer Res. Author manuscript; Available in PMC 2022 Feb 2. (PMC8808363; doi:10.1158/1078-0432.CCR-21-0343)
Supplement: Supplemental Figure 1 [file NIHMS1767618-supplement-Supplemental_Figure_1.docx]

**Supplemental Figure 1: Flow-chart of subsets of Mayo TNBC cohort grouped by analytical data (PD-L1 companion assays, RNA sequencing data and high-plex digital spatial profiling)**

TNBC subset in **corresponding TMA** with PD-L1 SP142 & 22C3 IHC Data

(N=231)

TNBC subset with PD-L1 SP142 IHC Data (**Whole-slide** FFPE)

(N=499)

**Mayo Early-Stage TNBC Cohort**

(N=605)

TNBC subset in **TMA** with PD-L1 SP142/22C3 IHC Data

+

**High-plex digital spatial profiling** (performed on TMA)

(N=184)

TNBC subset with PD-L1 SP142 Data (**Whole-slide** FFPE)

+

**RNA sequencing data** (from Whole-slide FFPE)

(N=234)
