# Supplementary material for: Characteristics and Spatially Defined Immune (micro)landscapes of Early-stage PD-L1–positive Triple-negative Breast Cancer
Source: Clin Cancer Res. Author manuscript; Available in PMC 2022 Feb 2. (PMC8808363; doi:10.1158/1078-0432.CCR-21-0343)
Supplement: Supplementary Table S1 [file NIHMS1767618-supplement-Supplementary_Table_S1.docx]

**Supplementary Table 1: Clinicopathologic Features of PD-L1+ TNBC in TMA scored by SP142 or 22C3 assays**

|  | **PD-L1 SP142 Assay* (TMA)** | | | **PD-L1 22C3 Assay** (TMA)** | | | | | |
| --- | --- | --- | --- | --- | --- | --- | --- | --- | --- |
|  | %IC+<1  (N=133) | %IC+≥1  (N=99) | p | CPS < 1  (N=125) | CPS ≥1  (N=106) | p | CPS < 10  (N=195) | CPS ≥10  (N=36) | p |
| **Menopausal Status** |  |  | 0.93 |  |  | 0.68 |  |  | 0.21 |
| Postmenopausal | 76 (57.1%) | 56 (56.6%) |  | 73 (58.4%) | 59 (55.7%) |  | 108 (55.4%) | 24 (66.7%) |  |
| Pre/Perimenopausal | 57 (42.9%) | 43 (43.4%) |  | 52 (41.6%) | 47 (44.3%) |  | 87 (44.6%) | 12 (33.3%) |  |
| **Age (y)** |  |  | 0.49 |  |  | 0.97 |  |  | 0.33 |
| ≥ 50 | 76 (57.1%) | 61 (61.6%) |  | 74 (59.2%) | 63 (59.4%) |  | 113 (57.9%) | 24 (66.7%) |  |
| < 50 | 57 (42.9%) | 38 (38.4%) |  | 51 (40.8%) | 43 (40.6%) |  | 82 (42.1%) | 12 (33.3%) |  |
| **Tumor Size** |  |  | 0.78 |  |  | 0.86 |  |  | 0.53 |
| Missing | 0 | 1 |  | 0 | 1 |  | 1 | 0 |  |
| ≤ 2.0 cm | 64 (48.1%) | 44 (44.9%) |  | 60 (48.0%) | 47 (44.8%) |  | 93 (47.9%) | 14 (38.9%) |  |
| 2.1-5.0 cm | 59 (44.4%) | 48 (49.0%) |  | 56 (44.8%) | 51 (48.6%) |  | 87 (44.8%) | 20 (55.6%) |  |
| ≥ 5.1 cm | 10 (7.5%) | 6 (6.1%) |  | 9 (7.2%) | 7 (6.7%) |  | 14 (7.2%) | 2 (5.6%) |  |
| **Axillary Nodal status** |  |  | 0.02 |  |  | 0.03 |  |  | 0.42 |
| Missing | 2 | 0 |  | 1 | 1 |  | 1 | 1 |  |
| 0 | 85 (64.9%) | 51 (51.5%) |  | 80 (64.5%) | 56 (53.3%) |  | 113 (57.9%) | 23 (63.9%) |  |
| 1-3+ | 29 (22.1%) | 27 (27.3%) |  | 27 (21.8%) | 29 (27.6%) |  | 47 (24.1%) | 9 (25.0%) |  |
| 4-9+ | 13 (9.9%) | 8 (8.1%) |  | 13 (10.5%) | 7 (6.7%) |  | 19 (9.7%) | 1 (2.8%) |  |
| ≥ 10+ | 4 (3.1%) | 13 (13.1%) |  | 4 (3.2%) | 13 (12.4%) |  | 15 (7.7%) | 2 (5.6%) |  |
| **Nottingham Grade** |  |  | 0.009 |  |  | 0.98 |  |  | 0.11 |
| Grade 1-2 | 12 (9.0%) | 1 (1.0%) |  | 7 (5.6%) | 6 (5.7%) |  | 13 (6.7%) | 0 (0.0%) |  |
| Grade 3 | 121 (91.0%) | 98 (99.0%) |  | 118 (94.4%) | 100 (94.3%) |  | 182 (93.3%) | 36 (100%) |  |
| **Ki-67 Proliferative Index** |  |  | 0.05 |  |  | 0.01 |  |  | 0.02 |
| Missing | 1 | 0 |  | 1 | 0 |  | 1 | 0 |  |
| ≤ 15% | 26 (19.7%) | 10 (10.1%) |  | 27 (21.8%) | 9 (8.5%) |  | 35 (18.0%) | 1 (2.8%) |  |
| 15.1-30% | 25 (18.9%) | 14 (14.1%) |  | 23 (18.5%) | 16 (15.1%) |  | 35 (18.0%) | 4 (11.1%) |  |
| ≥ 30% | 81 (61.4%) | 75 (75.8%) |  | 74 (59.7%) | 81 (76.4%) |  | 124 (63.9%) | 31 (86.1%) |  |
| **Stroma TIL scores (%)** |  |  | < 0.001 |  |  | < 0.001 |  |  | <0.001 |
| Median (Range) | 15  (1, 80) | 40  (1, 90) |  | 15  (1, 80) | 40  (5, 90) |  | 20  (1-80) | 40  (5, 90) |  |
| **Histologic Subtypes** |  |  | < 1e-04 |  |  | 0.09 |  |  | 0.01 |
| Apocrine | 10 (7.5%) | 2 (2.0%) |  | 6 (4.8%) | 6 (5.7%) |  | 12 (6.2%) | 0 (0%) |  |
| Invasive carcinoma NST | 97 (72.9%) | 63 (63.6%) |  | 93 (74.4%) | 66 (62.3%) |  | 139 (71.3%) | 20 (55.6%) |  |
| Medullary | 11 (8.3%) | 30 (30.3%) |  | 15 (12.0%) | 26 (24.5%) |  | 28 (14.4%) | 13 (36.1%) |  |
| Metaplastic | 15 (11.3%) | 4 (4.0%) |  | 11 (8.8%) | 8 (7.5%) |  | 16 (8.2%) | 3 (8.3) |  |
| **Surgery** |  |  | 0.20 |  |  | 0.37 |  |  | 0.84 |
| Lumpectomy | 69 (51.9%) | 43 (43.4%) |  | 64 (51.2%) | 48 (45.3%) |  | 94 (48.2%) | 18 (50.0%) |  |
| Mastectomy | 64 (48.1%) | 56 (56.6%) |  | 61 (48.8%) | 58 (54.7%) |  | 101 (51.8%) | 18 (50.0%) |  |
| **Adjuvant Radiation** |  |  | 0.99 |  |  | 0.20 |  |  | 0.31 |
| Missing | 18 | 20 |  | 24 | 14 |  | 34 | 4 |  |
| No | 48 (41.7%) | 33 (41.8%) |  | 38 (37.6%) | 43 (46.7%) |  | 65 (40.4%) | 16 (50.0%) |  |
| Yes | 67 (58.3%) | 46 (58.2%) |  | 63 (62.4%) | 49 (53.3%) |  | 96 (59.6%) | 16 (50.0%) |  |
| **Adjuvant Chemotherapy** |  |  | 0.045 |  |  | 0.29 |  |  | 0.06 |
| Missing | 15 | 19 |  | 20 | 14 |  | 31 | 3 |  |
| No | 44 (37.3%) | 19 (23.8%) |  | 37 (35.2%) | 26 (28.3%) |  | 57 (34.8%) | 6 (18.2%) |  |
| Yes | 74 (62.7%) | 61 (76.2%) |  | 68 (64.8%) | 66 (71.7%) |  | 107 (65.2%) | 27 (81.8%) |  |

*SP142 assays scored as % tumor-associated immune cells/tumor area; **22C3 assay scored as combined positive score (see Methods).
